# Lactation and Work: Managers’ Support for Breastfeeding Enhance Vertical Trust and Organizational Identification

**DOI:** 10.3389/fpsyg.2020.00018

**Published:** 2020-02-11

**Authors:** Ana María Lisbona, Miguel Bernabé, Francisco José Palací

**Affiliations:** Departamento de Psicología Social y Organizaciones, Facultad de Psicología, Universidad Nacional de Educación a Distancia, Madrid, Spain

**Keywords:** breastfeeding barriers, organizational identification, trust, organizational support, social identity, lactation

## Abstract

**Background:**

In working women, there are barriers when combining the mother and work role, especially during the breastfeeding period. Recent literature shows that improving organizational support increases trust performance via different domains (i.e., organizational identification) and that improving support for breastfeeding increases lactation rates and duration. Breastfeeding support in the workplace is one component that contributes to a mother’s ability to continue to breastfeed once she has returned to work. This is a Human Resource Management practice that facilitates a work–life balance. Working mothers have, at least, two roles: mother and worker and, when mothers return to work, they have to manage both identities. Is lactation a way to keep both identities connected? Is organizational support of breastfeeding a way to improve organizational identification? The aim of this paper is to analyze a hierarchical model to explain how managers and co-worker support to breastfeeding predict trust and organizational identity in a sample of Spanish working mothers (*N* = 1,028).

**Materials and Methods:**

To analyze the indirect effect, it was tested using a mediation model with PROCESS in two random samples and carried out structural equation modeling to confirm structural relationship in the proposed model.

**Results:**

Outcomes reveal effects of managers’ support to lactation and vertical trust in organizational identity but not in co-worker path.

**Conclusion:**

The findings suggest the manager’s role in maintaining trust from working women and create and maintenance organizational identification.

## Introduction

Breastfeeding rates are lower than health institutions recommend, and breastfeeding duration is sometimes shorter than mothers wish. To increase breastfeeding rates and help mothers achieve the lactation duration that both mothers and babies desire, lactation support is required.

From a social justice perspective, mothers who work need to be supported. There are women who need to work to provide for their family, and there are mothers who want to develop their professional life. All mothers have the right to give the best nutrition to their babies by breastfeeding them, and working mothers should also have the right to provide the best nutrition to their babies. To protect mothers who want to take care of their babies, maternity leave law is necessary, but there are countries where maternity leaves are not remunerated or are shorter than what the WHO recommends as an exclusive breastfeeding duration (6 months). The participants in this study work in Spain, where the maternity leave is 16 weeks, which is lower than what the WHO recommends.

Since there are mothers who want to breastfeed their babies without giving up their careers, organizations must support breastfeeding, both because it is fair and because if companies do not support mothers who combine employment and breastfeeding and make them choose between the two, the labor market may lose important talent sources. Furthermore, society may lose an opportunity to improve children’s and mother’s health through breastfeeding.

Because breastfeeding rates among working mothers are lower than those among mothers who are not employed (e.g., Greene et al., 2008), the main objective is to increase these rates. Previous research shows that a perceived lack of breastfeeding support from supervisors is related to an eightfold increase in women’s likelihood to discontinue exclusive breastfeeding, whereas perceived support for mothers returning to work predicts the continuation of exclusive breastfeeding (Burks, 2015; Spitzmueller et al., 2016). Improving organizational breastfeeding support should increase lactation rates in working mothers. These results show that it is more important to determine how mothers perceive support for breastfeeding in their own work than to assess the policies or norms that organizations have implemented because the mother’s perception is what matters. Although workplace lactation accommodations are important, it seems that the perception of support by the supervisor is more predictive of support than general organizational laws, spaces, or policies.

Working mothers or mothers who would like to work and breastfeed at the same time talk about the obstacles involved in combining both. It seems that public policies do not help to overcome these obstacles. Therefore, one way to remove barriers is to show how support for lactation in the workplace has positive benefits to organizations and to the society. This is a complex and ambitious objective. The aim of this paper is to analyze how workplace breastfeeding support predicts trust and organizational identification. One of our goals is to go beyond a social justice perspective and attempt to persuade organizational managers that supporting breastfeeding is beneficial to them because organizational support for breastfeeding is related to positive organizational outcomes. For this reason, we attempt to identify outcomes that connect organizational breastfeeding support with organizational processes or attitudes in the workplace that, in turn, have positive consequences for the organization and for work performance. Previous research has found some positive relationships between organizational breastfeeding support and organizational outcomes. Waite and Christakis (2015) found that if lactation support in the workplace is provided, working mothers’ job satisfaction is improved. In addition to these positive results, some researchers discuss positive consequences for the mother’s health. That is, organizational breastfeeding support may decrease depression based on the finding by Spitzmueller et al. (2016) of a negative relationship between organizational breastfeeding support and depressive symptoms. In the same way that a strategy to promote breastfeeding not only summarizes lactation benefits but also lists the dangers of not breastfeeding, we can review the risks to organizations if they do not support women who combine work and lactation. The literature shows negative outcomes or consequences for organizations that do not support breastfeeding. For instance, women who breastfeed sometimes experience more overload and conflicts between the needs of family and work than women who do not have to reconcile work and breastfeeding (Spitzmueller et al., 2016).

It seems obvious that to talk about breastfeeding is talk about healthy behavior, but the concept has also been applied to organizations. Then, we can introduce the Healthy And Resilient Organization (HERO) Model as a theoretical framework that join a healthy behavior, breastfeeding, with a healthy organization: those who support breastfeeding. A HERO organization is that organization that make systematic, planned, and proactive efforts to improve employees’ and organizational processes and outcomes (Salanova et al., 2012).

The Model is made up of three main interrelated components (Salanova et al., 2012): (a) healthy organizational resources and practices; (b) healthy employees, which refers to employees with positive psychological resources (e.g., organizational trust, self-efficacy, mental and emotional competences, organizational-based self-esteem, optimism, hope, and resilience), which are positively related to well-being (Acosta et al., 2012); and (c) healthy organizational outcomes. Two meta-analyzes summarize the benefits for mothers, and babies, of breastfeeding (Horta et al., 2007; Ip et al., 2007), so breastfeeding could also be considered within the block healthy employees, as organizational trust.

### Organizational Breastfeeding Support and Trust

A systematic review examining workplace lactation accommodations and their association with breastfeeding duration (Hilliard, 2017) concludes that the presence of a corporate lactation program, on-site child care, and return to work/telephone lactation consultation are consistently associated with breastfeeding until at least 6 months. Nonetheless, other breastfeeding accommodations (i.e., lactation spaces, lactation breaks, worksite lactation policies, and supervisor/co-worker support) were not consistently associated with breastfeeding duration. That study recommends promoting supervisor and co-worker support for improving lactation duration (Hilliard, 2017). Hence, we examine two domains, manager and co-worker support, from the Employee Perceptions of Breastfeeding Support Questionnaire (EPBS-Q) (Greene et al., 2008). This questionnaire analyzes five domains: organization, manager, co-worker, time, and physical environment.

Trust is a research topic that has been studied in different disciplines. For instance, from the biological perspective, trust and lactation have something in common: oxytocin. The relevance of the oxytocin hormone in the development of attachment and positive social relationships is the biological basis of trust (Acosta, 2017). From an organizational perspective, trust is a shared state that emerges from employees’ and teams’ interactions that creates a perception of organizations (Acosta, 2017, p.113). Furthermore, organizational trust is considered one of the key elements of the HERO Model. Specifically, it is a psychological construct included within the category of “healthy employees.”

In the organizational context, the literature (see Bachman and Zaheer, 2006) emphasizes two dimensions: horizontal and vertical trust. Vertical trust is an employee’s willingness to be vulnerable to the actions of the organization, whose behavior and actions he or she cannot control (Tan and Lim, 2009, p.46). As Acosta (2017) notes, trust is an important component in achieving individual and organizational goals. It is necessary to invest efforts to increase vertical trust, which can be done by increasing organizational breastfeeding support and specific manager support. Horizontal trust, or trust in one’s direct supervisor and in co-workers, represents employees’ willingness to be vulnerable to the actions of their direct supervisor, whose behavior and actions they cannot control (Acosta, 2017). If organizations increasing their vertical trust could also increase the achievement of goals, increasing horizontal trust could encourage innovation, and improve leadership effectiveness. Failure to do so reduces the likelihood of employees showing initiative (Acosta, 2017).

The Human Resources Management workers’ perceptions, like organizational breastfeeding support, could impact on workers attitude and behaviors (Manuti and Giancaspro, 2019) like organizational identification.

### Organizational Identification

Ashforth and Mael (1989) showed the usefulness of social identity theory (Tajfel and Turner, 1979) for the organizational scholarship. Since then, there have been many applications of the social identity theory to organizational phenomena. Social identification is the process by which a person’s group associations are internalized into his or her sense of self (so that the sense of self is defined in terms of “we” and “us” rather than “I” and “me”). That is, social identification refers to the readiness of an individual to perceive himself or herself as a representative of a particular group, which makes the individual perceive group characteristics as self-descriptive and leads him or her to adopt distinctive group norms as a guide for his or her own behavior (Ellemers et al., 2004). There are some foci of identification in a person’s life, including the role of being an employee and that of maternity. Mercer (2004) analyzes another theory in her work: the mid-range theory of maternal role attainment or becoming a mother. She describes four stages in the process of developing a maternal identity: (1) commitment, attachment, and preparation (pregnancy); (2) acquaintance, learning, and physical restoration (2–6 weeks following birth); (3) moving toward a new normal (2–4 months); and (4) achievement of maternal identity (around 4 months). The third stage of “moving toward a new normal” is often when mothers return to work and must manage both identities. Is lactation a way to keep both identities connected? One of our aims is to analyze how organizational support of breastfeeding is related to organizational identification.

Organizational identification is important because it can explain the process by which individuals engage with groups and organizations. If organizational identification is strong, it prompts deindividuation, fostering the internalization of group (organizational) norms and attributes (Blader and Tyler, 2009). Tyler and Blader (2000, 2003) hold that social identity is necessary for understanding the psychological basis of people’s engagement with groups, organizations, and societies. A meta-analytic review of social identification and health in organizational contexts (Steffens et al., 2017) found a mean weighted positive association between organizational identification and health (defined as well-being and absence of stress). Workplaces should do everything possible to encourage organizational identification. Thus, the research question is whether employees’ perception of support improves organizational identification. In an organizational context, studies (e.g., Edwards and Peccei, 2010; Marique et al., 2013) show a positive relationship between perceived organizational support and organizational identification. Therefore, we expect that employees’ perceived support for breastfeeding will also have a positive relationship with organizational identification.

Some studies have found a positive relationship between organizational trust and organizational identification (e.g., Farooq et al., 2014). The present study distinguishes between two dimensions of employees’ perceived support for breastfeeding (manager and co-worker support) because we expect that each dimension is related to each type of trust (vertical and horizontal). This leads us to define a first hypothesis at the organizational level involving managers and vertical trust:

Hypothesis 1: Manager support for breastfeeding has an indirect effect on organizational identification through vertical trust.

The other hypothesis is at the teamwork level and involves co-workers’ support for breastfeeding and horizontal trust. In both hypotheses, the dependent variable is organizational identification.

Hypothesis 2: Co-worker support for breastfeeding has an indirect effect on organizational identification through horizontal trust.

By adding both hypotheses, we can define the proposed model.

## Materials and Methods

### Design and Participants

The study design was cross-sectional. The participants (*N* = 1,028) were working women who were employed in Spain. A total of 97% of the participants reported that their children had been breastfed. The participants’ average age was 37 years (*SD* = 6.17), and most of them were married (66%). Furthermore, 59.5% of the participants had a university education. Regarding socio-labor variables, 70% worked full time. More information about the sample can be found in Table 1. To prevent random capitalization, the sample was divided into two. Sample 1 (*n*_1_ = 503) had an average age of 37 years (*SD* = 6.45), and 98.1% reported that their children had been breastfed. Of sample 1, 66% of the participants reported being married and 58% reported having a university education, and 70% worked full time. Sample 2 (*n*_2_ = 525) presented similar values. The average age was 37.3 (*SD* = 5.88), 65% were married, 69% worked full time, and 97% reported breastfeeding their children.

**TABLE 1:** Information about population who have participated in this study.

	*N*	%
**Type of workday**		
Full working days	561	54.6
Split shift	142	13.8
Part time	213	20.7
Freelance	88	8.6
Other type	24	2.3
**Working hours**		
8 h/day	791	79
4 h/day or less	237	21
**Professional sector**		
Administration	101	10
Customer assistance	15	1.5
Accounting, banking, and finance	11	1.1
Education and teaching	83	8.2
Business	11	1.1
Management	50	5
Technical career, architecture, and engineering	85	8.4
Medicine	96	9.5
Health services	230	22.8
Community services	56	5.6
Technician and operators	60	6
Sales and commerce	85	8.4
Others	145	14.4
**Type of breastfeeding**		
Exclusive	156	20.3
Mixed	614	79
**Number of children**		
At least 1 son	566	56.2
2 children	380	37.7
3 children	68	6.7
4–6 children	14	1.4

### Variables and Questionnaires

#### Organizational Identification

The organizational identification scale (Lisbona et al., 2006) (α = 0.71) is a Spanish adaptation that provides a review of the main scales of social and organizational identification conducted by Mael and Ashforth (1992) and Haslam (2001). It includes the considerations of van Knippenberg and van Schie (2000) and Grice et al. (2002) to evaluate organizational identification using seven items. An example of an item is: “I feel personally insulted when someone criticizes my organization.” The response scale was a Likert-type 5-point scale.

#### Organizational Trust

Organizational trust was measured using two dimensions: vertical trust (α = 0.91) and horizontal trust (α = 0.87), from the HERO questionnaire (Salanova et al., 2012). Vertical trust (four items) corresponds to an adaptation of the vertical trust scale from Huff and Kelley (2003). An example item is, “In this organization, subordinates have enormous trust in supervisors and in management.” Horizontal trust (four items) is an adaptation of the McAllister (1995) questionnaire (1995). An example item is, “In this organization, we can share our ideas, emotions and hopes.” The response scale was a Likert-type 7-point scale measuring the degree of agreement or disagreement regarding perceived trust in the organization.

#### Organizational Support for Breastfeeding

Organizational support measures manager support (12 items) and co-worker support (6 items) from the EPBS-Q (see Appendix I; Greene et al., 2008). The response scale was a Likert-type 4-point scale measuring the degree of agreement or disagreement regarding perceived social support behaviors for breastfeeding in an organizational setting. An example item for the manager support scale (α = 0.88) is: “My manager helps me combine breastfeeding with work.” An example item for the co-worker support scale (α = 0.83) is: “My colleagues cover some of my tasks if I need time to breastfeed.”

### Procedure

Data were collected using the QUALTRICS platform. Snowball sampling was followed to collected data. The questionnaires were distributed by researchers of research group in Breastfeeding and Perinatal Psychology of IMIENS. These distributed the questionnaires through the Facebook Group of the UNED breastfeeding specialist course. In turn, the teachers and students of the course collaborated in this distribution. The participants were informed of the objectives of the research, the voluntary nature, and the anonymous and confidential use of the data. After acceptance of informed consent, the participants completed the questionnaires.

### Statistical Analysis

Analysis of the internal consistency of the scales was conducted using Cronbach’s alpha to evaluate the validity of the questionnaires. Higher values (α > 0.70) are indicators of a good level of internal consistency (Nunnally and Bernstein, 1994). Statistically significant relationships were analyzed through descriptive analysis (mean and standard deviation) and Pearson’s product–moment correlation with a significance level of *p* < 0.001. *t* tests were conducted for the different analyses of the random samples generated before the regression analysis. To study the effects established in the hypotheses and to analyze the indirect effect on the dependent variable, the bootstrap procedure developed by Preacher and Hayes (2004) was used. According to this procedure, the indirect effects are significant when *p* < 0.05 is 95% of confidence intervals. To determine the strength of the mediating effect, the macro MEDIATE of SPSS developed by Preacher et al. (2007) was used. This procedure conducts multiple regression analysis to check indirect and direct effects on the dependent variable. To confirm the structural relationship, structural equation modeling (SEM) was conducted to study the adequacy of the proposed model. A good fit of the model is indicated when the chi-square is significant, the relation between χ^2^/*df* is in the range (2–3); the goodness-of-fit index, comparative fit index (CFI), and normed fit index (NFI) are >0.90; and root mean square error of approximation (RMSEA) is between (0.05 and 0.08) (Lau et al., 2009). To accept the mediating effect, the Sobel test was used at *p* < 0.05 with a critical value corresponding to *z*/2 = 1.96 that allows it to be considered a mediating effect on the dependent variable. The statistical program SPSS 24 was used for statistical analysis and internal consistency. The SEM analysis was conducted out by means of AMOS. For the hierarchical analysis of mediation, the macro of Preacher and Hayes (2004) was used in SPSS 24.

## Results

The matrix of Pearson correlations can be observed in Table 2. As shown, the *t* test does not show significant differences between the mean values for sample 1 (*n*_1_ = 503) and sample 2 (*n*_2_ = 525). The scales’ mean values do not differ for organizational identification (*F* = 0.00; *t* test = 0.86; *p* = 0.38), manager support (*F* = 0.04; *t* test = 1.18; *p* = 0.23), co-worker support (*F* = 0.12; *t* test = −0.32, *p* = 0.74), VT (*F* = 0.24; *t* test = 0.41; *p* = 0.67), and horizontal trust (*F* = 0.17; *t* test = −0.60; *p* = 0.54). Mean values and standard deviations for both samples can be found in Table 2.

**TABLE 2:** Cronbach values, descriptive (mean and standard deviation), and Pearson correlation values.

		Total	Sample 1	Sample 2					

Variables*	α	*M(SD)*	*M_1_(SD_1_)*	*M_2_(SD_2_)*	(1)**	(2)	(3)	(4)	(5)
1. OI	0.71	2.92 (1.13)	2.96 (1.14)	2.88 (1.12)	–	0.23	0.20	0.40	0.20
2. MS	0.88	2.62 (1.04)	2.68 (1.04)	2.57 (1.04)		–	0.46	0.39	0.41
3. CS	0.83	2.80 (0.88)	2.78 (0.89)	2.81 (0.88)			–	0.24	0.47
4. VT	0.91	3.79 (1.27)	3.81 (1.28)	3.77 (1.26)				–	0.41
5. HT	0.87	4.59 (1.01)	4.57 (1.03)	4.61 (0.99)					–

*OI, organizational identification; MS, manager support; CS, co-worker support; VT, vertical trust; HT, horizontal trust. **All the correlations are statistically significant (*p* < 0.005).

As Table 3 shows, hierarchical regression analysis indicates a direct effect of vertical trust on organizational identification in both randomized samples (*n*_1_: β = 0.329, *SE* = 0.060, *t* = 5.476, *p* = 0.000; *n*_2_: β = 0.322, *SE* = 0.055, *t* = 5.819, *p* = 0.000). However, a direct effect is not observed in another horizontal trust on organizational identification (*n*_1_: β = 0.046, *SE* = 0.081, *t* = 0.579; *p* = 0.563, *n*_2_: β = −0.002, *SE* = 0.072, *t* = −0.040, *p* = 0.867). Regarding indirect effects of organizational support, as shown in Table 3, sample 1 manager support has an indirect effect by vertical trust on organizational identification [β = 0.166, *SE* = 0.043, 95% CI (0.087–0.255)]. This indirect effect is replicated in sample 2 [β = 0.146, *SE* = 0.036, 95% CI (0.080–0.221)].

**TABLE 3:** Direct effect on organizational identification.

	β	*SE*	*t*	*p* value
**Sample 1**				
Vertical trust	0.329	0.060	5.476	0.000
Horizontal trust	0.046	0.081	0.579	0.563
Manager support	−0.009	0.079	−0.118	0.906
Co-worker support	0.144	0.094	1.59	0.112
**Sample 2**				
Vertical trust	0.322	0.055	5.819	0.000
Horizontal trust	−0.002	0.072	−0.040	0.967
Manager support	0.029	0.071	0.413	0.680
Co-worker support	0.164	0.088	1.861	0.063

Co-worker support does not show an indirect effect in any of the mediators (see Table 4). The Omnibus Test found that the indirect effects of manager support are statistically significant for the two samples (*n*_1_: β = 0.050, *SE* = 0.016, CI 0.023–0.088; *n*_2_: β = 0.053; *SE* = 0.020, CI 0.023–0.102). Hence, manager support and vertical trust predict organizational identification in both randomized groups (*n*_1_: *R*^2^ = 0.443, *F* = 13.01, *p* = 0.000; *n*_2_: *R*^2^ = 0.439, *F* = 14.95, *p* = 0.000).

**TABLE 4:** The indirect effect of organizational support on organizational identification.

	β	*SE*	95% CI
**Vertical trust**			
Manager support (MS_1_)	0.166	0.043	[0.087–0.255]
Manager support (MS_2_)	0.146	0.036	[0.080–0.221]
Co-workers support (CS_1_)	0.011	−0.055	[−0.056–0.083]
Co-workers support (CS_2_)	0.020	−0.033	[−0.044–0.091]
**Horizontal trust**			
Manager support (MS_1_)	0.013	0.026	[−0.038–0.067]
Manager support (MS_2_)	−0.000	0.013	[−0.032–0.028]
Co-workers support (CS_1_)	−0.015	0.032	[−0.029–0.058]
qqCo-workers support (CS_2_)	−0.015	0.040	[−0.084–0.078]

*1, sample 1; 2, sample 2.*

**FIGURE 1:**
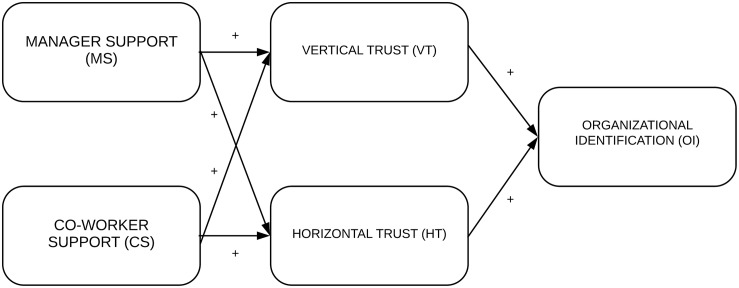
Proposed theoretical model.

**FIGURE 2:**
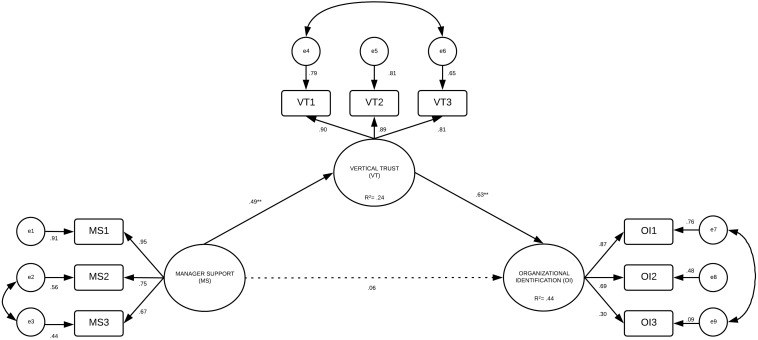
Mediation model of manager support, vertical trust on organizational identification. **p<0.005.

To confirm the structural relationship, a theoretical model was tested on the total sample. Two models were analyzed. The output of Model 1 (Figure 1) showed values that did not meet the criteria [χ^2^/*df* = 3.77, *p* = 0.000, CFI = 0.941, NFI = 0.948, PCFI = 0.64, parsimony comparative of fit index (PCFI) = 0.05]. Subsequently, the model was respecified, taking into consideration the previous results for the randomized samples (Hypothesis 1). That is, it was tested for a direct effect (Model 2) and an indirect effect (Model 3, see Figure 2) of manager support and vertical trust on organizational identification. The results show that Model 3 (χ^2^/*df* = 2.09, *p* = 0.002, CFI = 0.993, NFI = 0.986, PCFI = 0.48, RMSEA = 0.03) had better adjustment rates than Model 2 (χ^2^/*df* = 2.27, *p* = 0.034, CFI = 0.992, NFI = 0.987, PCFI = 0.28, RMSEA = 0.04). Therefore, manager support has an indirect effect on organizational identification via vertical trust. To test the mediational role of vertical trust on manager support–organizational identification, the Sobel test (*z* = 4.74, *SE* = 0.023, *p* < 0.000) was conducted and showed that the effect of manager support on organizational identification decreased upon the addition of vertical trust to the path, which is in line with the criteria of Baron and Kenny (1986). That is, the third path (*c* = 0.39, *SE* = 0.039, *p* = 0.000) decreases (*c*′ = 0.06, *SE* = 0.020, *p* = 0.278) when vertical trust is in the model effect of manager support on organizational identification.

## Conclusion

There is a gap between organizational psychology research and between breastfeeding research, mainly when combined lactation and work is required. The study examined whether employees’ perceived support for breastfeeding improves trust and organizational identification. To support this analysis, the study proposed two hypotheses. One focused on the organizational level and considered perceived manager support for breastfeeding and its indirect effect on organizational identification through vertical trust. The second focused on the teamwork level and measured co-workers’ support for breastfeeding and its indirect effect on organizational identification through horizontal trust.

Hierarchical regression analysis and structural model analysis confirm Hypothesis 1 but not Hypothesis 2. From these results, we can conclude that perceived manager support for breastfeeding is more important for improving organizational identification and vertical trust, whereas co-worker support is not related to horizontal trust or organizational identification. Therefore, it is necessary to encourage organizations to provide through management to mothers who breastfeed. Hilliard (2017) found similar results regarding peer support, meaning that supervisor/co-worker support seems not to be relevant for breastfeeding duration.

In this line, it is possible that for structural barriers (i.e., the absence of national regulation to promote breastfeeding at work), the help provided by the head is more salient as an instrumental support. That is, manager support “buffers” the stress impact associated with job demands in front of co-worker support. This it could be understood as a trade-off, which increases affective commitment with the organization as social exchange theory (Saks, 2006) argue. An alternative explanation is that managers could reduce work-to-family conflict in contrast with co-workers because the first source could reassign tasks or reduce role expectations, which could relax role conflict (i.e., mother–worker).

We believe in the need to support lactation in working mothers and attempt to demonstrate to organizational managers that supporting breastfeeding in the workplace is fair and may improve some organizational processes, such as the trust or organizational identity. This is the first step in a more complex study of the conflict between breastfeeding and work. This study has some limitations, such as considering only organizational identification and not considering other objective variables such as performance. However, showing how a supporting breastfeeding is beneficial to managers and organizations is a powerful outcome that might persuade managers to do the right thing.

Every day, there are more organizations that put their effort in human resources management towards getting healthier employees who, in turn, will achieve healthier results for the organization (better performance) through healthy practices, following the HERO model. We suggest organizational breastfeeding support as one of those healthy practices that encourage healthy employees, also favoring another key aspect in the management of healthy organizations: the balance between work and personal life.

Finally, a limitation of the study to consider is that it is a cross-sectional study, so the results should be considered adequately without inferring causality of the variables.

## Data Availability Statement

The datasets generated for this study are available on request to the corresponding author.

## Ethics Statement

The studies involving human participants were reviewed and approved by the National University of Distance Education. The patients/participants provided their written informed consent to participate in this study.

## Author Contributions

All authors have contributed significantly to the article and have given their consent to appear in it, and were responsible for data collection coordinated by FP. MB led the data analysis and elaborated proposal in coordination with the other authors. AL supervised all the process and coordinated the drafting and preparation of the submitted manuscript.

## Conflict of Interest

The authors declare that the research was conducted in the absence of any commercial or financial relationships that could be construed as a potential conflict of interest.

## Appendix I

Employee Perceptions of Breastfeeding Support Questionnaire (EPBS-Q; Greene et al., 2008): Manager and Co-worker support scales.

1. My manager would support me breastfeeding or pumping breast milk at work.
2. My manager would help me combine breastfeeding and work.
3. My manager would think I couldn’t get all my work done if I needed to take breaks for breastfeeding or pumping breast milk.
4. I would feel comfortable speaking with my manager about breastfeeding.
5. My manager says things that make me think he/she supports breastfeeding.
6. I feel my manager would view breastfeeding as an employee’s personal choice.
7. My manager would consider it part of his/her job to help me combine breastfeeding and work.
8. My manager would think less of workers who choose to breastfeed or pump breast milk at work.
9. My manager would make sure my job is covered if I needed time for breastfeeding or pumping breast milk.
10. My manager would make change my work schedule to allow me time for breastfeeding or pumping breast milk.
11. My manager would help me deal with my workload so I could breastfeed or pump breast milk at work.
12. My manager would be embarrassed if I spoke with him/her about breastfeeding.
13. My co-workers would think less of workers that choose to breastfeed or pump breast milk at work.
14. I would feel comfortable speaking with my co-workers about breastfeeding.
15. My co-workers say things that make me think they support breastfeeding.
16. My co-workers would change their break times with me so that I could breastfeed or pump breast milk.
17. My co-workers would cover my job duties if I needed time for breastfeeding or pumping breast milk.
18. My co-workers would be embarrassed if I spoke with them about breastfeeding.
